# Sodium glucose co-transporter 2 (SGLT2) inhibition via dapagliflozin improves diabetic kidney disease (DKD) over time associatied with increasing effect on the gut microbiota in db/db mice

**DOI:** 10.3389/fendo.2023.1026040

**Published:** 2023-01-26

**Authors:** Jiajia Wu, Yan Chen, Huinan Yang, Leyi Gu, Zhaohui Ni, Shan Mou, Jianxiao Shen, Xiajing Che

**Affiliations:** ^1^ Department of Nephrology, Renji Hospital, School of Medicine, Shanghai Jiao Tong University, Shanghai, China; ^2^ Division of Gastroenterology and Hepatology, Key Laboratory of Gastroenterology and Hepatology, Ministry of Health, State Key Laboratory for Oncogenes and Related Genes, Shanghai Institute of Digestive Disease, Renji Hospital, School of Medicine, Shanghai Jiao Tong University, Shanghai, China; ^3^ School of Energy and Power Engineering, University of Shanghai for Science and Technology, Shanghai, China

**Keywords:** dapagliflozin, diabetes kidney disease (DKD), *Muribaculaceae*, *Lactobacillus*, bile acid, therapeutic targets

## Abstract

**Background:**

The intestinal microbiota disorder gradually aggravates during the progression of diabetes. Dapagliflozin (DAPA) can improve diabetes and diabetic kidney disease(DKD). However, whether the gut microbiota plays a role in the protection of DAPA for DKD remains unclear.

**Methods:**

To investigate the effects of DAPA on DKD and gut microbiota composition during disease progression, in our study, we performed 16S rRNA gene sequencing on fecal samples from db/m mice (control group), db/db mice (DKD model group), and those treated with DAPA (treat group) at three timepoints of 14weeks\18weeks\22weeks.

**Results:**

We found that DAPA remarkably prevented weight loss and lowered fasting blood glucose in db/db mice during disease progression, eventually delaying the progression of DKD. Intriguingly, the study strongly suggested that there is gradually aggravated dysbacteriosis and increased bile acid during the development of DKD. More importantly, comparisons of relative abundance at the phylum level and partial least squares-discriminant analysis (PLS-DA) plots roughly reflected that the effect of DAPA on modulating the flora of db/db mice increased with time. Specifically, the relative abundance of the dominant Firmicutes and Bacteroidetes was not meaningfully changed among groups at 14 weeks as previous studies described. Interestingly, they were gradually altered in the treat group compared to the model group with a more protracted intervention of 18 weeks and 22 weeks. Furthermore, the decrease of *Lactobacillus* and the increase of *norank_f:Muribaculaceae* could account for the differences at the phylum level observed between the treat group and the model group at 18 weeks and 22 weeks.

**Conclusion:**

We firstly found that the protective effect of DAPA on DKD may be related to the dynamic improvement of the gut microbiota over time, possibly associated with the impact of DAPA on the bile acid pool and its antioxidation effect.

## Introduction

Diabetic kidney disease (DKD) is one of the most common chronic kidney diseases globally, with growing incidence and prevalence (1), as 30% to 40% of patients with diabetes will have complications such as DKD. Chronic inflammation, insulin resistance, poor glycemic control, and oxidative stress have been reported to be driving forces in DKD (2, 3). However, therapies based on these mechanisms have limited effects; more researches about DKD pathogenesis are essential and may provide new insights into treating DKD. Intriguingly, the gut microbiota as a novel intervention for diabetes and its complications, such as DKD, is now attracting more and more attention (4–6).

The gut microbiota has a symbiotic relationship with the host, involving energy metabolism, regulating the gut barrier, and maintaining immune responses (7). Many studies have consistently demonstrated (8–12) that changes in the composition of gut microbiota regulate the development of diabetes by inducing continuous low-grade inflammation and mediating the therapeutic effects of some type 2 diabetes mellitus(T2DM)drugs (13–15).

Sodium glucose co-transporter 2 inhibitors (SGLT2) are the oral treatments for T2DM, with a widely accepted mechanism by reducing the renal threshold of glucose (16). Recently, clinical studies have shown that SGLT2 inhibitors can remarkably prevent DKD progression and the onset of end-stage renal disease independent of lowering glucose (17, 18) and, thus, as SGLT2 inhibitors, Canagliflozin and Dapagliflozin (DAPA) have been used to delay the development of DKD (19). Nevertheless, the underlying mechanisms of SGLT2 inhibitors still need to be fully addressed. For example, some animal studies have shown that SGLT2 inhibitors could reduce albuminuria in db/db mice. While a few recent studies showed SGLT2 inhibitors for 10 weeks did not see any changes in albuminuria using db/db mice, explaining that this phenomenon may be associated with the timing of administration and mild renal histological injury (20, 21). Given that, in this study, we started to administrate DAPA at 6 weeks and set three timepoints at early and late stages of DKD to fully present the renal protective effect of DAPA.

Although Canagliflozin has been reported to reconstruct the gut microbiota in mice with chronic kidney disease (22), there are few and controversial studies about the effects of DAPA on the fecal microbiota of diabetes. Notably, to the best of our knowledge, no study has reported the association between the protection of DAPA on DKD and the gut microbiota. Two studies in 2018 indicated that DAPA could modify the fecal microbiota in animal models of diabetes after 6 or 8 weeks of intervention, accompanied by changing the F/B ratio and microbiota diversity (23, 24). Interestingly, one study in 2020 showed that DAPA did not affect the ratio of F/B and microbiota diversity in a type 2 diabetic rat model at a 1 mg/kg/day dose for 4 weeks (14), implicating the effects of DAPA enhanced possibly over time. Besides, two studies have shown that DAPA, administered for 6 days or 6 weeks, can control blood glucose well without changing colonic or fecal microbiota in the diabetes model, as previous studies described (25, 26). More importantly, one human study reported that DAPA administration did not affect the fecal microbiota in T2DM patients treated with metformin (27). The inconsistency of these results may be related to differences in the length (6 days- 8 weeks) and dose of drug intervention, and the drug combination choice. In short, the DAPA had minor or no effects on the gut microbiota in db/db mice on the condition of the administration period for 6 days - 8 weeks, as most previous studies described. Noteworthily, the administration time of 6 days - 8 weeks is not enough for studying the role of DAPA in DKD associated with its regulation of the gut microbiota. As DAPA is generally continuously used in the long-period treatment of DKD in clinical practice and SGLT2 inhibitors, especially DAPA, are often administrated for 10-12w or longer time rather than 6 days – 8 weeks or less time for the treatment of DKD in db/db mice (21, 28–30); future studies should explore whether DAPA as a novel therapy for DKD can regulate the gut flora and we assumed that the prolonged intervention of DAPA has further benefits. To our knowledge, we firstly suggest that the protective effect of DAPA on DKD may be related to the improvement of the gut microbiota and investigate the impacts of DAPA on the gut flora in the DKD mice over time.

## Methods

### Animals and tissue collection

All animal research was approved by the Institutional Animal Ethics Committee of Renji Hospital. The animal experiment ethics approval number is m20170324. We purchased 5-week-old male C57BL/6 mice and BKS.Cg-Dock7m +/+ Leprdb/J (db/db) mice from SLAC Laboratory Animal Co., Ltd. (Shanghai, China). We housed all mice in a light- and temperature-controlled facility with free access to water and food. After one week of adaptation, we set three groups: control group (C57BL/6 mice administrated with the same volume of physiological saline as the treatment group), model group (db/db mice administrated with the same volume of physiological saline as the treatment group) and treat group (db/db mice treated with DAPA [1.0 mg/kg/day, AstraZeneca, Cambridge, UK]) respectively at three timepoints of 14 weeks,18 weeks and 22 weeks. DAPA mixed in the drinking water and the same volume of physiological saline were administrated by oral gavage once daily. The body weight and fasting blood glucose levels of the mice were measured every 2 weeks during the treatment period, and the urinary albumin to creatinine ratio (uACR) was measured every 4 weeks. To obtain the pathological gold standard, mice were euthanized at different time points of 8, 12, or 16 weeks following the treatment. Immediately afterwards, the kidneys, intestines, and blood were collected. We pathologically confirmed renoprotective effects of DAPA and then sent the guts of the corresponding mice with DKD improvement to be sequenced.

### Biochemical analysis

We used the Albumin Creatinine Ratio Assay Kit (ab241018) to measure mice’s urine albumin concentration and urine creatinine concentration. A Liquid Urea Nitrogen Reagent Set and Creatinine Assay kit (Nanjing Jiancheng Bioengineering Institute, China) was used to measure BUN and plasma creatinine levels.

### Histopathology analyses of renal tissue

We preserved the renal tissues in 10% neutral formalin and embedded them in 10% paraffin. Sections (5 µm thick) were subjected to periodic acid–Schiff (PAS). The glomerulosclerosis index (GSI) was adopted to quantify lesions on PAS-stained paraffin sections. One renal pathologist assessed over 50 glomeruli randomly chosen from each mouse in a blinded manner under ×400 magnification.

### DNA extraction and sequencing

We used the E.Z.N.A.^®^ soil DNA Kit (Omega Bio-Tek, Norcross, GA, U.S.) to extract total microbial genomic DNA per sample. We used 1.0% agarose gel electrophoresis and a NanoDrop^®^ ND-2000 spectrophotometer (Thermo Scientific Inc., USA) to determine the quality and concentration of DNA, which were kept at -80 °C before the subsequent use. The hypervariable region V3-V4 of the bacterial 16S rRNA gene was amplified, with all samples amplified in triplicate. We extracted the PCR product from 2% agarose gel and used the AxyPrep DNA Gel Extraction Kit (Axygen Biosciences, Union City, CA, USA) and Quantus™ Fluorometer (Promega, USA) to purify and quantify the PCR product, which was then pooled in equimolar amounts, and paired-end sequenced on an Illumina MiSeq PE300 platform (Illumina, San Diego, USA) complied with instructions by Majorbio Bio-Pharm Technology Co. Ltd. (Shanghai, China).

### Data analysis

We carried out the bioinformatic analysis of the gut microbiota using the Majorbio Cloud platform (https://cloud.majorbio.com). Based on the OTUs information, we calculated alpha diversity indices, including ace richness and Shannon index with Mothur v1.30.1. The similarity among the microbial communities in different samples was determined by β-diversity using the Mothur program.

### Statistical analyses

Data are expressed as mean ± standard deviation (SD). ANOVA was used to evaluate the statistical significance among multiple groups. The statistical significances between the two groups were calculated by Student’s unpaired t-test. The significant differences of genera were assessed using Wilcoxon rank-sum test. The differences were considered statistically significant at P<0.05. Partial least squares-discriminant analysis (PLS-DA) plots of Bray–Curtis dissimilarity were performed to visualize the group differences.

## Result

### Effects of DAPA on fasting blood glucose and body weight in db/db mice

**Figure 1:**
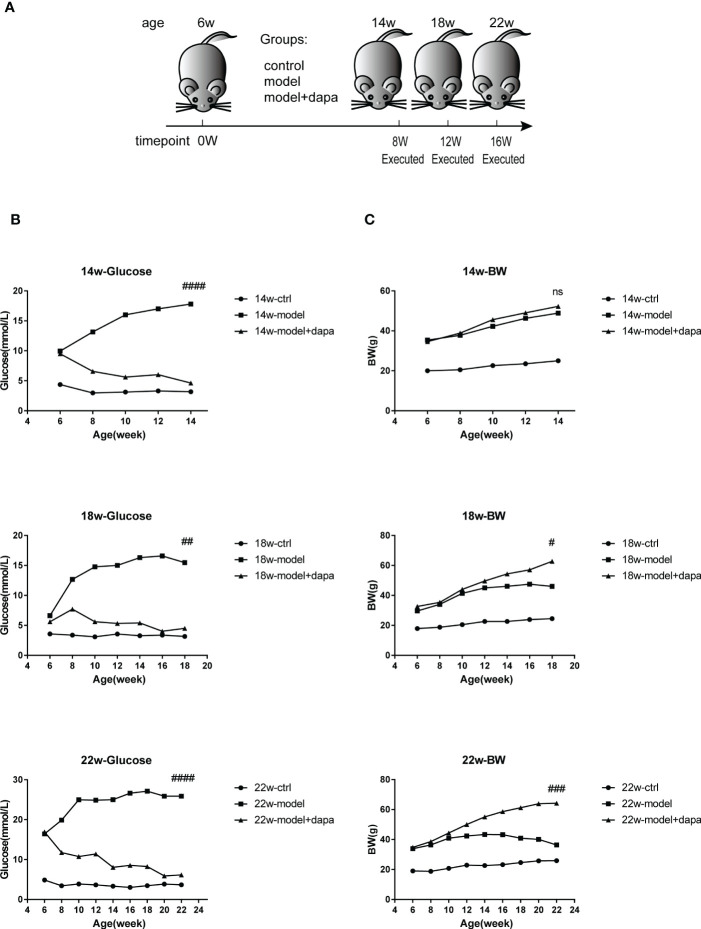
DAPA effectively controls fasting blood glucose and body weight in db/db mce. **(A)** the Experimental design for mice. Briefly, six-week-old male nondiabetic db/m and diabetic db/db mice were randomly divided into three group (db/m + physiological saline mice (ctrl group), db/db + physiological saline mice (model group), and db/db model mice + DAPA (model +DAPA group)) and administrated by oral gavage once daily with physiological saline or 1.0 mg/kg/day DAPA, finally executed respectively at 14w (14 weeks), 18w (18 weeks), and 22w(22 weeks). **(B)** Fasted blood glucose levels respectively from 6w to 14w, 18w, and 22w. **(C)** Changes in body weight respectively from 6w to 14w, 18w, and 22w. Statistical significance was calculated using ANOVA with Tukey's test. N=4/group. ^#^p < 0.05, ^##^p < 0.01, ^###^p < 0.001, ^####^p < 0.0001, ns, not significant for model group vs model + DAPA group at the same time point. ctrl: the control group.

To evaluate the effects of DAPA on fasting blood glucose and body weight in db/db mice (**Figure 1A**), one well-known spontaneous diabetic nephropathy model, we measured the changes in fasting blood glucose and body weight over time. The study revealed that db/db mice initially had higher serum glucose than db/m mice. In contrast, a dramatic and consistent decrease in serum glucose was observed in db/db mice after 1 mg/kg DAPA treatment for 8, 12, and 16 weeks (**Figure 1B**). The mice in the treat group gained weight with survival time. The average body weight at 18 and 22 weeks in the treat group were higher than those in the other groups (**Figure 1C**), consistent with the result that DAPA could restore weight loss at the late stage of diabetes (23). Notably, the model group did not begin to show a trend of weight loss at 14 weeks (**Figure 1C**), so it may be reasonable that there were no significant differences in the body weight of mice between the model and treat group at 14 weeks. Collectively, DAPA had a therapeutic effect on hyperglycemia and could significantly reduce weight loss in db/db mice.

### Dapagliflozin effectively slows the progression of DKD in db/db mice

**Figure 2:**
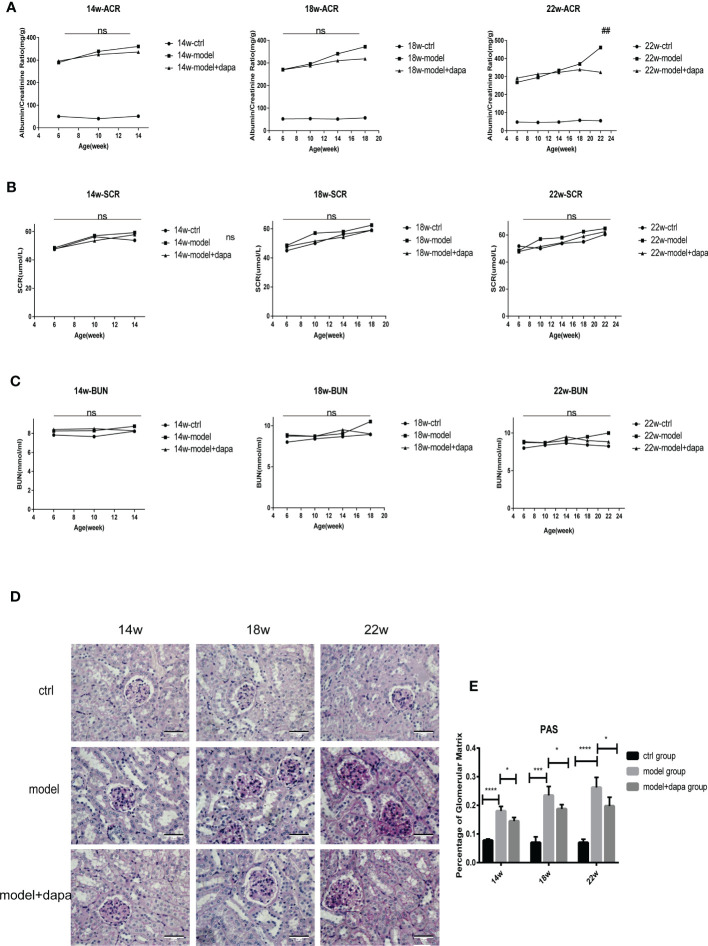
DAPA effectively slows the progression of DKD in db/db mice. **(A–C)** Albumin to creatinine ratio (ACR), scr, and bun levels were determined every 4 weeks in the mice of three groups throughout 14 weeks (14w), 18 weeks (18w), and 22 weeks (22w). **(D, E)** Periodic acid-Schiff (PAS) staining analysis of the histopathological changes among three groups at 14w, 18w, and 22w. original magnification, x400. Scale bars, 50 µm. Data in **(D)** were quantified **(E)**. n = 4/group. Statistical significance was calculated using ANOVA with Tukey's test. ^*^p < 0.05, ^***^p < 0.001, ^****^p < 0.0001. ns, not significant for the indicated comparison. Ns in **(A)** is for comparison between the model group and model + DAPA group, ns in **(B, C)** is for comparison among three groups. ^**^p < 0.01 for model group vs mode; + DAPA group.

Our results showed that db/db mice had markedly higher uACR levels than db/m mice as expected, the rise of which was a typical manifestation of renal impairment, indicating that early-stage DKD occurred in 6-week-old db/db mice (31). Importantly, DAPA administration at a dose of 1 mg/kg restrained the uACR levels in the treat group at 22 weeks, the late stage of DKD reflected by the remarkable weight loss(P<0.05) (**Figure 2A**), interestingly, no significant changes in the uACR levels was seen but with improvements in pathology after DAPA treatment at 14 and 18 weeks in line with one recent study indicating that the uACR level changes can be seen with more severe renal histological injury in the late stage of DKD. No significant differences were observed in serum creatinine and urea nitrogen levels among the three groups during the observation period (**Figures 2B, C**). Periodic acid-Schiff staining of renal tissue showed that db/db mice in the model group exhibited increased renal structure damage, such as glomerular mesangial matrix expansion and mesangial hyperplasia compared to the control group; the pathologies mentioned above were remarkably alleviated in the treatment group compared to the model group at the same time point of 14, 18, 22 weeks (**Figures 2D, E**), indicating that DAPA treatment successfully protected from kidney damage.

### Dapagliflozin gradually modulates the overall structure of the gut microbiota in db/db mice

**Figure 3:**
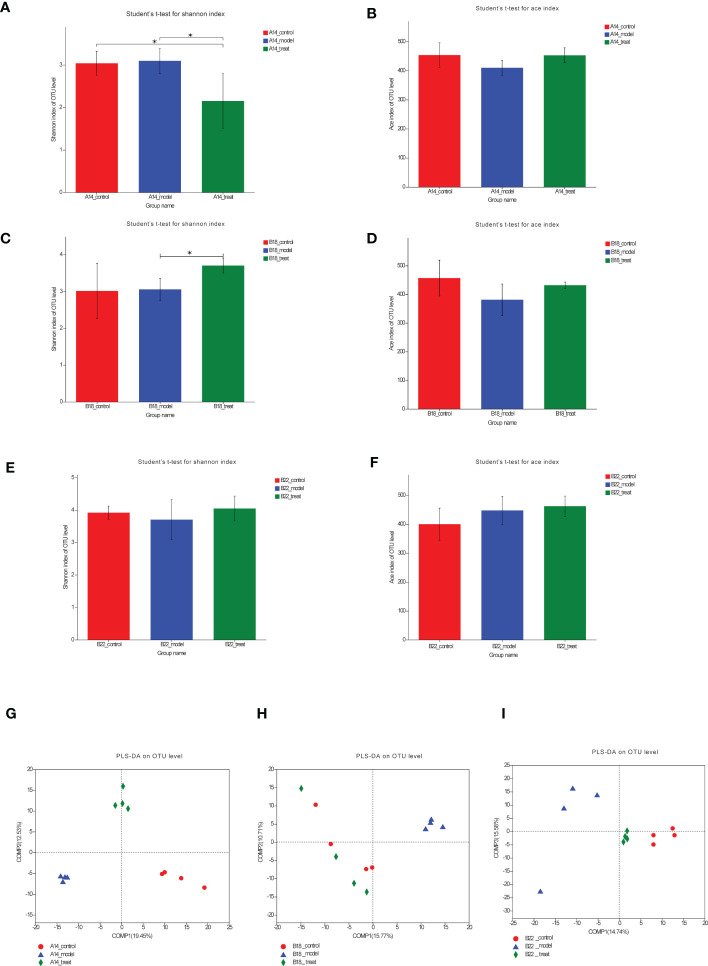
DAPA gradually modulates the overall structure of the gut microbiota in db/db mice. **(A, C, E)** Shannon diversity and ace richness **(B, D, F)** of fecal samples across three groups OUT at 14 weeks (14w), 18 weeks (18w), and 22 weeks (22w). **(G–I)** PLS-DA of OTU-level Bray-Curtis at 14w, 18w, and 22w. n = 4/group. Data are expressed as mean ± SEM. Statistical significance was calculated using ANOVA with Tukey's test. ^*^p < 0.05. PLS-DA, partial least squares-discriminant analysis. The treat group means model + DAPA group.

To compare the α-diversity and β-diversity of the gut microbiota composition among the three groups at three timepoints, we subjected fecal samples of three groups at different timepoints to 16S rRNA analysis. Bacterial community diversity was measured by the Shannon index and ace index, which indicates bacterial community richness. The Shannon index of the DAPA-treated group was significantly lower than that of the model group (**Figure 3A**); however, the ace index of the treat group had an increasing but not significant trend compared with the control group at 14 weeks (p=0.056) (**Figure 3B**). As we expected, the Shannon index was found to be higher in the treat group compared to the model group at 18 weeks with the therapy time prolonged (**Figure 3C**), although the ace index did not differ between the two groups (**Figure 3D**). At 22 weeks, there were increasing but not significant trends in the Shannon index and ace index after DAPA treatment (**Figures 3E, F**), which may result from the variances in the degree of disease development in mice within groups. Besides, although the indexes of richness and diversity failed to show a notable change between the model group and the control group at 14, 18, and 22 weeks, partial least squares-discriminant analysis (PLS-DA) plots of Bray–Curtis dissimilarity at three timepoints showed that the dots of the model group were not close to the dots of the control group, indicating there are distinct differences in the structure of intestinal flora between the two groups (**Figures 3G–I**). Remarkably, although the PLS-DA at 14 weeks suggested that the gut microbiota composition of the three groups was far apart, the plots of the treat group were closer to the fields of the control group than the model group to the control group at 18 weeks and 22 weeks (**Figures 3G–I**), which may be driven by a more extended intervention of DAPA. The heat maps of Bray–Curtis distance presented similar findings (**Supplementary Figures 1A–C**). Taken together, these results suggested that DAPA could dramatically alter the structure of the gut microbiota in a time-dependent manner.

### The effect of dapagliflozin to dramatically restore the dysbiosis of db/db mice at the phylum levels enhanced over time

**Figure 4:**
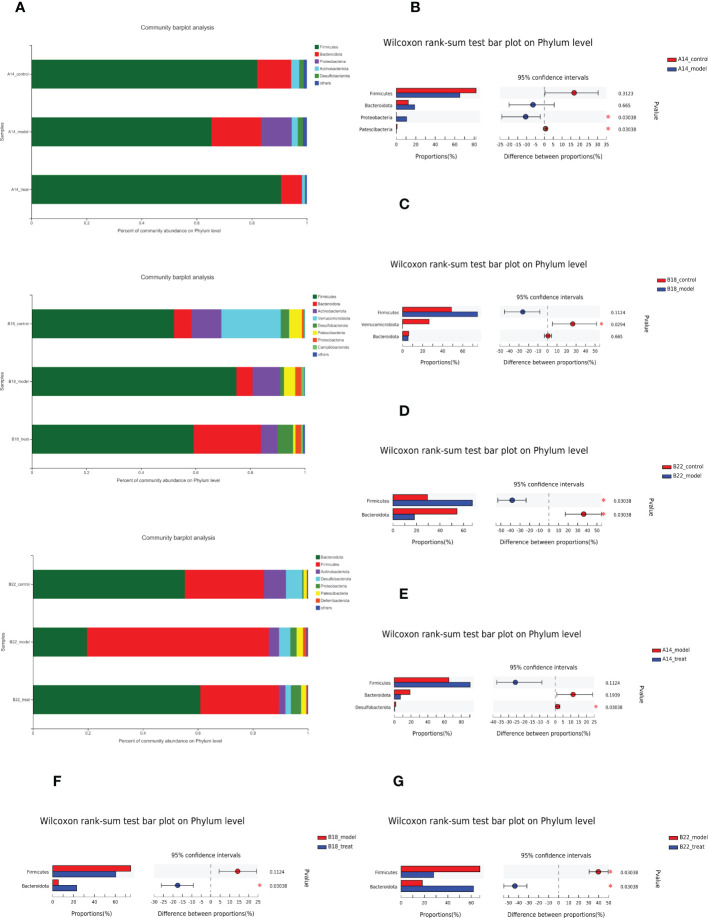
The effect of DAPA to dramatically restore the dysbiosis of db/db mice at the phylum levels enhanced over time. **(A)** Relative abundance of bacterial phyla, indicating changes in microbiota composition among three groups at 14 weeks (14w), 18 weeks (18w), and 22 weeks (22w). **(B–D)** Presentation of phyla with significant changes between the control group and model group at 14w, 18w, and 22w apart from dominant Firmicutes and Bacteroidota. **(E–G)** Presentation of phyla with significant changes between model group and model + DAPA group at 14w, 18w, and 22w besides dominant Firmicutes and Bacteroidota. N=4/group. Statistical significance was calculated using Wilcoxon rank-sum test. ^*^p < 0.05. The treat group means model + DAPA group.

As shown in **Figure 4A**, although Firmicutes and Bacteroides still accounted for the most significant proportion in the overall structure of intestinal flora from three groups at different time points, the relative abundance of some bacteria changed a lot. Specifically, compared to the control group, the model group had a higher quantity of Proteobacteria and a relatively lower abundance of Patescibacteria at 14 weeks (**Figure 4B**), while at 18 weeks, the relative abundance of Verrucomicrobiota decreased (**Figure 4C**), accompanied by an increased but not significant trend of Firmicutes. As the molding time lengthens, the model group was characterized by remarkably elevated levels of Firmicutes together with decreased abundance of Bacteroidetes compared to the control group at 22 weeks (**Figure 4D**), indicating the gut microbiota of db/db mice being gradually disordered over time and Bacteroidetes and Firmicutes may have crucial impacts on the DKD during the disease progression.

Further comparison of the bacterial taxa revealed differences between the treat and model groups. The treat group showed a lower abundance of Desulfobacterota at 14 weeks (**Figure 4E**). Interestingly, DAPA treatment increased the relative abundance of Bacteroidota at 18 weeks (**Figure 4F**) and then consistently rescued flora disorder of DKD by reducing the relative abundance of Firmicutes and further increasing the relative abundance of Bacteroidota at 22 weeks (**Figure 4G**), which strongly suggested that the effect of DAPA on the gut microbiota of db/db mice enhanced with time.

### Dapagliflozin consistently remodels the gut microbiota composition of db/db mice at the genus level

**Figure 5:**
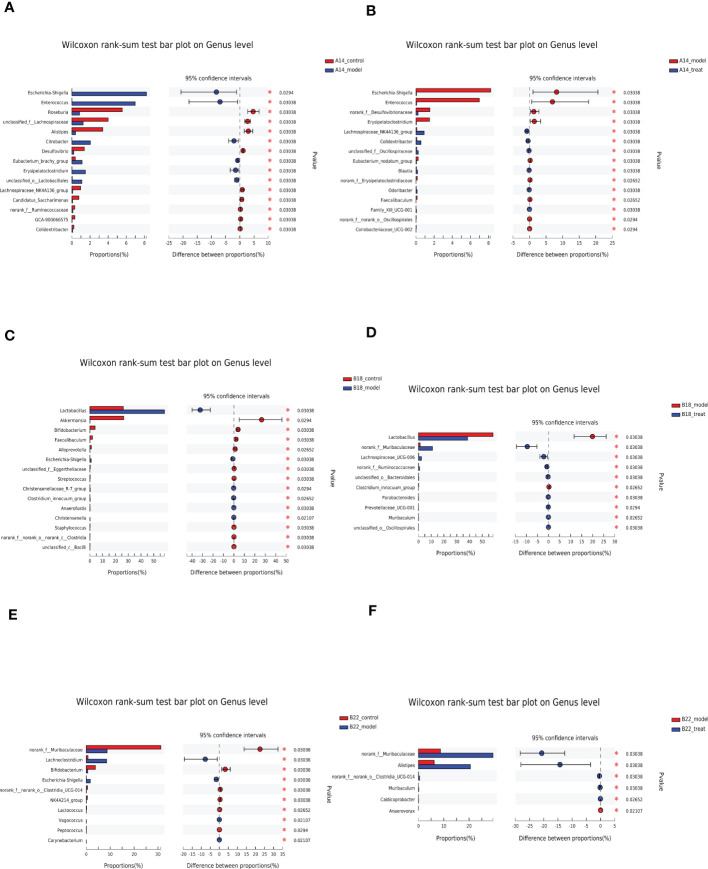
DAPA consistently remodels the gut microbiota composition of db/db mice at the genus level. **(A, C, E)** Significantly changed top 15 genera sorted by their abundances between the control group and model group at 14 weeks (14w), 18 weeks (18w), and 22 weeks (22w). **(B, D, F)** Significantly changed top 15 genera sorted by their abundances between the model group and model + DAPA group at 14w, 18w, and 22w. n=4/group. Statistical significance was calculated using Wilcoxon rank-sum test. ^*^p < 0.05. The treat group means model + DAPA group.

To further investigate the changes in the microbiota signature among groups, the analysis was carried out at the genus level (**Figures 5A–F** and **Supplementary Figures 2A–C**). Compared with the control group, *Escherichia-Shigella*, *Enterococcus*, *Citrobacter*, etc., were strikingly elevated. In contrast, *Roseburia*, *unclassified_f:Lachnospiraceae*, *Alistipes*, etc., in the DKD model group were markedly reduced at 14 weeks (**Figure 5A**). Interestingly, the results showed that the expansion of detrimental intestinal bacteria at 14 weeks was restrained after DAPA treatment. Specifically, *Escherichia-Shigella*, *Enterococcus*, *norank_f:Desulfovibrionaceae*, *Eubacterium_nodatum_group*, etc., decreased. Besides, *Lachnospiraceae_NK4A136_group*, *Colidextribacter*, *unclassified_f:Oscillospiraceae*, *Blautia, Odoribacter, etc.* increased in the treat group compared with the model group at 14 weeks (**Figure 5B**). With the disease progression, at 18 weeks, in addition to *Escherichia-Shigella, Lactobacillus* also significantly increased in the model group compared to the control group (**Figure 5C**), which was strikingly restored by DAPA administration (**Figure 5D**). Besides, beneficial bacteria such as *Akkermansia*, *Bifidobacterium*, *Faecalibaculum, Alloprevotella*, etc., in the model group decreased compared with the control group (**Figure 5C**). Notably, DAPA administration greatly enriched beneficial *norank_f:Muribaculaceae* apart from *Lachnospiraceae_UCG-006*, *norank_f:Ruminococcaceae*, *unclassified_o:Bacteroidales*, *Parabacteroides*, *Prevotellaceae_UCG-001*, *Muribaculum*, *unclassified_o:Oscillospirales*, etc. compared with the model group (**Figure 5D**).

At 22 weeks, in the model group, *Escherichia-Shigella* and *Lachnoclostridium* increased along with *Lactobacillus* having an increasing tendency (**Figure 5E**). On the other hand, *norank_f:Muribaculaceae*, *Bifidobacterium*, *norank_f:norank_o:Clostridia_UCG-014*, *NK4A214_group*, and *Lactococcus* decreased compared with the control group (**Figure 5E**). Greatly decreased *norank_f:Muribaculaceae* resulting from DKD can be rescued by DAPA administration which also boosted *Alistipes*, *norank_f:norank_o:Clostridia_UCG-014*, *Muribaculum*, *Caldicoprobacter*, etc. and reduced *Anaerovorax.* Notably, the expansion of *Lactobacillus* was restricted after DAPA administration, although there is no significant difference compared with the model group (**Figure 5F** and **Supplementary Figure 2C**).

### Bile acid may be associated with CKD progression and one of the DAPA intervention target

No significant KEGG pathway was enriched using PICRUSt analysis for the fecal microbiome among three groups at 14 weeks, which may be due to the early stage of DKD and short DAPA intervention (**Supplementary Figure 3A**). However, the pathway of primary bile acid biosynthesis was significantly upregulated in the DKD group at 18 weeks and *bilophila* tended to rise in the DKD group at 22 weeks (**Supplementary Figures 3B, C**). DAPA intervention tended to reverse the bile acid change, although it was not significant (**Supplementary Figures 3B, C**).

Overall, these results suggested that DAPA increasingly and consistently prevented DKD-driven dysbiosis at the genus level, phylum level and overall structure of the gut microbiota, which may be associated with the effect of DAPA on bile acid pool and its antioxidation effect.

## Discussion

We presented evidence that daily administration of db/db mice with DAPA was sufficient to prevent diabetes-induced weight loss and hyperglycemia, further easing the DKD, indicated by strikingly reduced proteinuria and deposition of mesangial matrix showed by Periodic acid-Schiff staining. Although studies showed that DAPA could reduce body weight, we observed that the weight of the treat group gradually increased, while the db/db mice had different degrees of weight loss at 18 weeks and 22 weeks, which are consistent with the previous research (23, 26, 32). In this regard, weight gains after DAPA intervention may reflect the improvement in disease status in db/db mice.

To explore the characterization of dysbiosis implicated in the progression of DKD and further determine whether DAPA administration could restore the structure of the gut microbiota or not, we performed 16s rRNA sequencing of fecal samples.

Regarding the first purpose, we firstly showed the increasing gut microbiota disorder and bile acid in DKD. Specifically, at the phylum level, the results at 14 weeks showed that the abundance of Proteobacteria, containing many harmful bacteria, was highly elevated, accompanied by diminished beneficial Patescibacteria without changing Firmicutes and Bacteroidota, while at 18 weeks, the probiotic Verrucomicrobiota widely spread in the healthy human intestine suppressing the inflammation process declined and the Firmicutes has an increasing but not significant trend. Interestingly, as the DKD progressed, a further increase in the proportion of Firmicutes occurred along with a decrease in Bacteroidota at 22 weeks which is common in diabetes and cardiac diseases. Besides, this result was consistent with a previous study showing that the increased F/B ratio was not always linked with a fat phenotype (23). Likewise, similar alterations were observed at the genus level. In the model group, conditional pathogenic bacteria such as *Escherichia-Shigella* expanded; however, beneficial bacteria such as *Roseburia*, *unclassified_f:Lachnospiraceae*, *Alistipes*, *Akkermansia*, and *Bifidobacterium* diminished. The *Lactobacillus* belonging to the Firmicutes has an increasing trend, and the *norank_f:Muribaculaceae* belonging to the Bacteroidetes showed a decreasing trend, which is more evident at 22 weeks, namely the late stage of the disease. Taken together, this trend at different levels was more pronounced at 22 weeks and 18 weeks than at 14 weeks consistent with KEGG pathways enrichment results. Therefore, there was a developing dysbiosis and bile acid accumulation in the progression of DKD. Our study raised the possibility that regulating the gut microbiota could be a promising strategy for DKD therapy. Based on that, it is reasonable to hypothesize that the effect of DAPA on the gut microbiota could mediate its protective role.

More importantly, consistent with the second hypothesis, our study showed that DAPA influenced the composition of intestinal flora, and this effect was enhanced with the prolongation of intervention time. On the whole, the α diversity was reduced in the treat group compared with the model group at 14 weeks; although this did not meet our expectations, but this is consistent with a previous study in which microbial diversity declined after the same short period of DAPA administration for improving diabetes and vascular dysfunction (23). Interestingly, with the intervention going on, α diversity increased significantly at 18 weeks and also showed an increasing trend at 22 weeks. Besides, the PLS-DA results further demonstrated the protective effect of DAPA on the gut microbiota over time. At the phylum level, the initial DAPA response at 14 weeks appeared to be driven by minor differences across phyla rather than noticeable changes of Firmicutes and Bacteroidetes due to short intervention explaining no significant KEGG pathways enrichment, and later the response at 18 weeks and 22 weeks were driven by significant changes of certain bacteria from the two predominant phyla. In our study, after DAPA treatment for 16 weeks, Bacteroidetes increased, and Firmicutes decreased at 22 weeks, suggesting DAPA rescued the overall changes in the gut microbiota of DKD. Bacteroidetes and Firmicutes take on responsibilities in improving glucose metabolism and lipid metabolism with enzymes such as α-glucosidases and α-amylases and hold the balance on gut microbiota due to their large proportion (33). Moreover, the bigger F/B ratio in the gut flora was reported to be associated with more pro-inflammatory cytokines and stronger insulin resistance (34, 35). Therefore, the regulation of DAPA on Bacteroidetes and Firmicutes might be necessary for its hypoglycemic effects.

The effects of DAPA on the gut flora at the genus level were consistent with the whole. At 14 weeks, DAPA did not enrich beneficial bacteria such as *Bifidobacteriaceae* but confined the boom of pathobionts, including *Escherichia-Shigella* and *Enterococcus*, related to impairing the intestinal barrier and therefore worsening kidney disease by activating the innate immune system in line with the previous study with the same short intervention (14). Other protective *Lachnospiraceae_NK4A136_group*, *Colidextribacter* abundant in healthy controls (36), *unclassified_f:Oscillospiraceae*, *Blautia producing acetic acid (* 37) and *Odoribacter* associated with succinate consumption (8) expanded.

At both 18 and 22 weeks, DAPA consistently reversed the abundance changes of *Lactobacillus* and *norank_f:Muribaculaceae*, reflecting the principal change of firmicutes and Bacteroides in DKD mice. In agreement with previous studies (10, 35), the percentage of *Lactobacillus* increased in the diabetic model. Our study showed that DAPA could reverse the trend, strongly implicating that *Lactobacillus* may play a role in metabolic disorders emerging in the progression of DKD as *Lactobacillus* was recently reported to induce or maintain low-grade inflammation (10, 14, 38, 39). *Muribaculaceae*, also named the S24-7 and belonging to Bacteroidetes, was the dominant bacterium at 18 and 22 weeks in treat group, in line with previous studies (13, 14). The abundance of *Muribaculaceae* has been implicated in predicting the levels of short-chain fatty acids (SCFAs), such as propionate production, and partially mediated the exercise-driven prevention of obesity (40–43). Other DAPA-enriched genera also have potential benefits. For example, *Alistipes* is a genus of Bacteroidetes mainly producing SCFAs such as acetate and propionate (44). Besides, *Ruminococcus* can inhibit the production of ROS by making ursodeoxycholic acid (45). Taken together, these results indicated that DAPA could remodel the gut microbiota to increase the production of SCFAs and perform the anti-inflammatory property, therefore ameliorating kidney damage.

Notably, the abundance of *Lactobacillus* seemed to be always negatively associated with the quantity of *norank_f:Muribaculaceae* in the DKD model group and DAPA intervention group. Thinking about the association between Firmicutes and Bacteroides at 18 weeks and 22 weeks, by searching PubMed online articles, one recent report in 2022 concluded that acids produced by *lactobacilli* inhibited the growth of commensal *Lachnospiraceae* and *Muribaculaceae* (46). Besides, similar to the microbial alteration pattern of negative correlations in our study, studies showed that treatment with intervention hugely reversed the increased F/B ratio, which is the hallmark of dysbiosis, accompanied by a striking downregulation of *Lactobacillus* belonging to Firmicutes (47, 48). One possible explanation for the phenomenon is that bile acid favored genus belonging to Firmicutes, such as BA (bile acid)-resistant *Lactobacillus* (35), and reducing the bile acid pool may remove the competitive advantage of *Lactobacillus spp* (47–49). Interestingly, our study also showed increased bile acid in the later stage of DKD reflected by the enriched primary bile acid biosynthesis at 18 weeks and increased *bilophila* at 22 weeks in the model group, at least partly explaining the expanded Firmicutes in the model group in our study. Besides, DAPA intervention in our study tended to reverse the bile acid change, possibly resulting in the reversed F/B ratio. Moreover, the antioxidant property of specific intervention may restrain oxygen availability and further compromise the bloom of facultative anaerobe species such as *Lactobacilli (* 47, 50). Indeed, the disorder of bile acid metabolism exists in DKD patients (10, 51). The change in bile acids induced by medication has also been reported to be correlated with better clinical outcomes, thereby improving the metabolic health of DKD (52, 53). In addition, DAPA could function by reducing the generation of ROS (54). Therefore, DAPA-driven modulation in Bacteroidetes and Firmicutes may root in the impacts of DAPA on the bile acid pool and its antioxidation effect. However, whether DAPA delayed the progression of DKD *via* regulating the gut microbiota such as *norank_f:Muribaculaceae* and *Lactobacillus* and especially bile acid, needs to be further verified in future research.

In conclusion, DAPA remarkably protected the progression of DKD. Its impact on modulating the flora of db/db mice at 14 weeks was roughly in line with the previous study, and our study uniquely presented its increased effects reflected by the consistent changes at phylum and genus levels, which may be associated with its regulation on bile acid at 18 and 22 weeks. To the best of our knowledge, we firstly suggest that the protective effect of DAPA on DKD may be related to the improvement of the gut microbiota, possibly linked with bile acid in a time-dependent manner, which provides more solid evidence that prolonged DAPA intervention enhances the regulation of dysbacteriosis in DKD and renders a new target for DKD therapy.

## Data availability statement

The data presented in the study are deposited in the SRA database, accession number PRJNA923132.

## Ethics statement

The animal study was reviewed and approved by the Institutional Animal Ethics Committee of Renji Hospital.

## Author contributions

JW and YC designed the experiments, analyzed the data, and wrote the manuscript. HY carried out the experiments. LG and ZN provided critical materials. SM revised the manuscript. XC and JS supervised the project. All authors have read and approved the final version of the manuscript.
