# Supplementary figures and images for: Sodium glucose co-transporter 2 (SGLT2) inhibition via dapagliflozin improves diabetic kidney disease (DKD) over time associatied with increasing effect on the gut microbiota in db/db mice
Source: Front Endocrinol (Lausanne). 2023 Jan 26;14:1026040. doi: 10.3389/fendo.2023.1026040 (PMC9908601; doi:10.3389/fendo.2023.1026040)

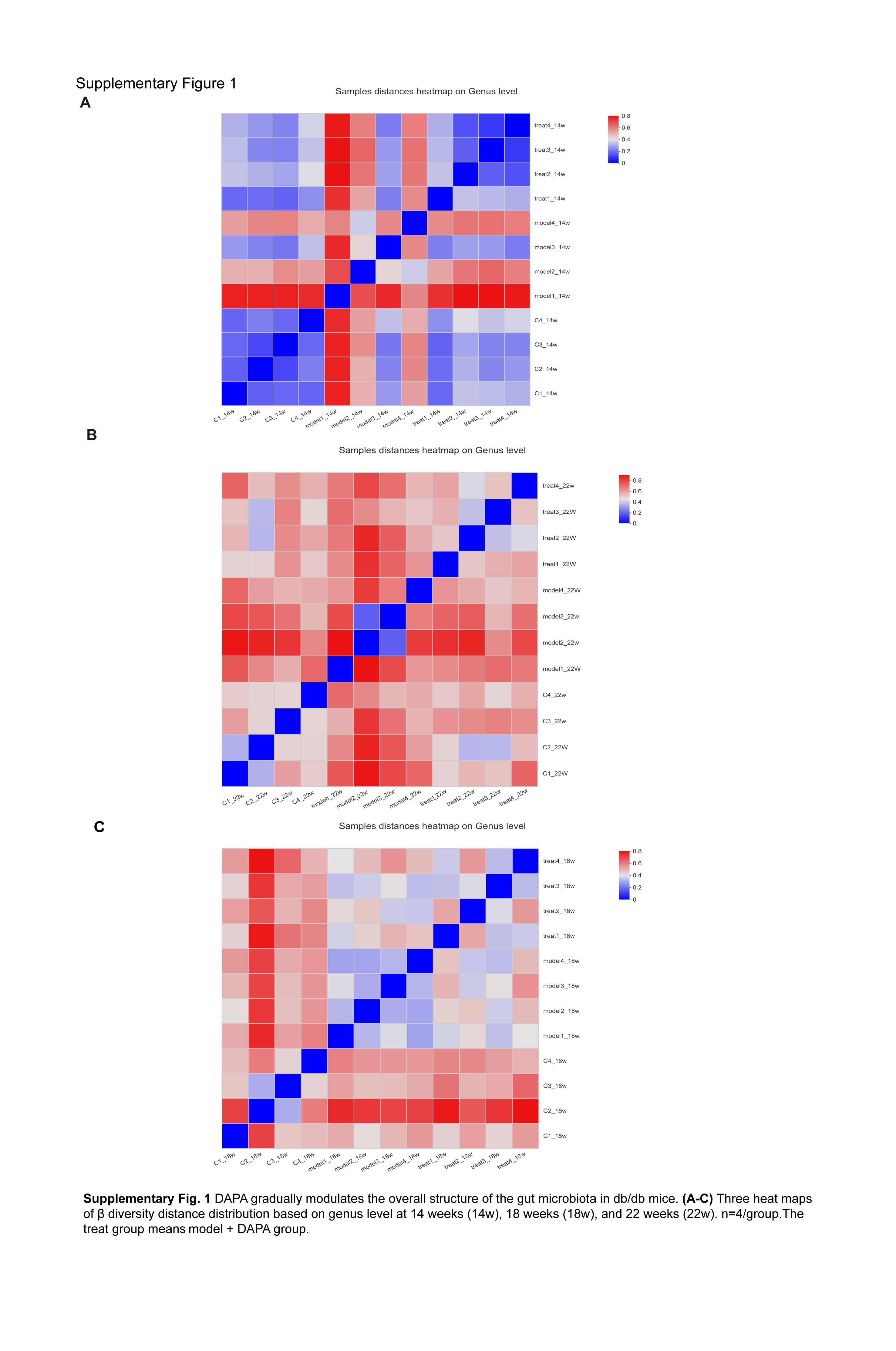

Supplement: Supplementary file 1 [file Image_1.tif]

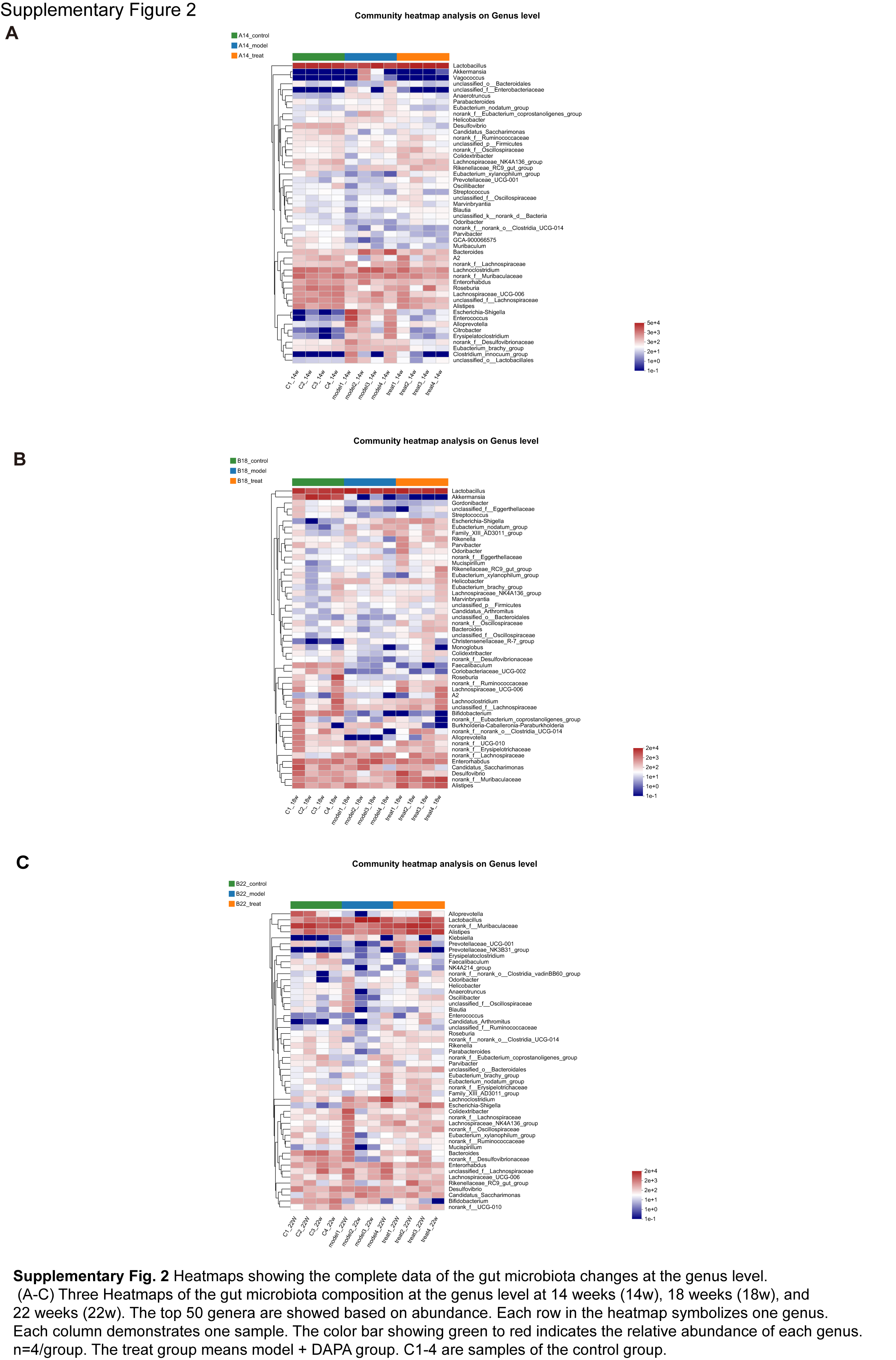

Supplement: Supplementary file 2 [file Image_2.tif]

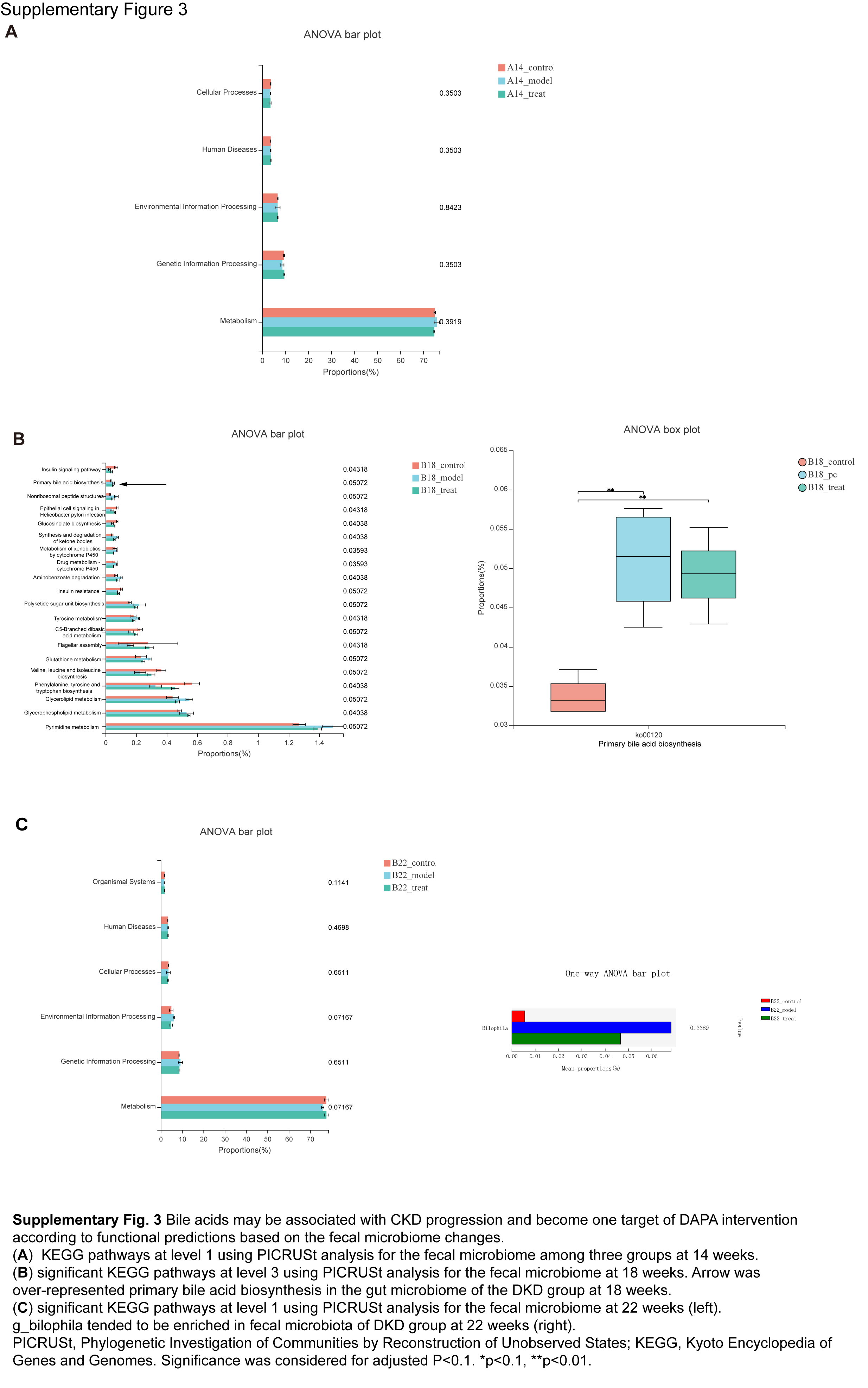

Supplement: Supplementary file 3 [file Image_3.tif]
